# Central Apparatus, the Molecular Kickstarter of Ciliary and Flagellar Nanomachines

**DOI:** 10.3390/ijms22063013

**Published:** 2021-03-16

**Authors:** Zuzanna Samsel, Justyna Sekretarska, Anna Osinka, Dorota Wloga, Ewa Joachimiak

**Affiliations:** Laboratory of Cytoskeleton and Cilia Biology, Nencki Institute of Experimental Biology, Polish Academy of Sciences, 3 Pasteur Street, 02-093 Warsaw, Poland; z.samsel@nencki.edu.pl (Z.S.); j.sekretarska@nencki.edu.pl (J.S.); a.osinka@nencki.edu.pl (A.O.); d.wloga@nencki.edu.pl (D.W.)

**Keywords:** axoneme, central pair microtubules, *Chlamydomonas*, *Trypanosoma*, PCD, male infertility

## Abstract

Motile cilia and homologous organelles, the flagella, are an early evolutionarily invention, enabling primitive eukaryotic cells to survive and reproduce. In animals, cilia have undergone functional and structural speciation giving raise to typical motile cilia, motile nodal cilia, and sensory immotile cilia. In contrast to other cilia types, typical motile cilia are able to beat in complex, two-phase movements. Moreover, they contain many additional structures, including central apparatus, composed of two single microtubules connected by a bridge-like structure and assembling numerous complexes called projections. A growing body of evidence supports the important role of the central apparatus in the generation and regulation of the motile cilia movement. Here we review data concerning the central apparatus structure, protein composition, and the significance of its components in ciliary beating regulation.

## 1. Introduction

Typical motile cilia and flagella (CFs) are several to several dozen micrometers long, microtubule-based organelles formed at the surface of eukaryotic cells, from protists to humans. These two homologous structures are early evolutionarily inventions, proposed to be present in the last eukaryotic common ancestor (LECA) [1,2]. During the evolution, CFs were lost by organisms from several lineages whose reproduction has become independent of water (Dikarya and most seed plants). In animals (Metazoa), these organelles have undergone functional and structural speciation giving rise to, besides CFs, immotile sensory cilia, and motile nodal cilia [1]; all these organelles differ from each other in terms of ultrastructure and functions. In vertebrates, typical motile CFs are assembled by the epithelial cells lining the respiratory tract, brain ventricles, and oviduct as multiple organelles (from several dozen up to ~250 per cell, [3,4,5] or as a single long flagellum by sperm cells. Nodal cilia are present in cells of the embryonic node as a single organelle [6]. Immotile sensory cilia can be present as multiple structures (e.g., the number of immotile olfactory cilia varies from several in mammals up to ~100 in zebrafish [7,8,9]), but most often are assembled as a single structure (primary cilium) by non-dividing or differentiated cells [10,11].

**Figure 1:**
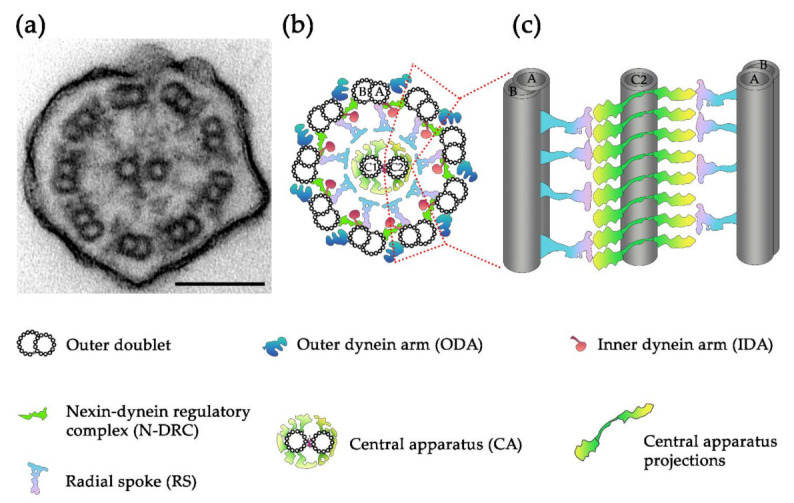
Structure of motile cilia/flagella (CFs): (**a**) a transmission electron microscopy (TEM) cross-section of a *Tetrahymena thermophila* cilium, bar = 100 nm; (**b**) the organization of axonemal complexes across the CF circumference, C1 and C2—central microtubules 1 and 2, respectively, A and B—tubules A and B of the outer doublet; (**c**) the longitudinal organization of the outer doublet radial spokes (RS) and central apparatus (CA) projections showing possible interactions between these structures; for the simplicity only C2 microtubule is shown. Note that for clarity, other outer doublet complexes (ODA, IDA and N-DRC) are not shown.

The functional specialization of cilia correlates with the differences in their ultrastructure. In all CF types, the skeleton (an axoneme) is composed of nine peripherally positioned microtubular doublets, but only in motile cilia, both typical and nodal; these microtubules are docking sites for multiprotein complexes [12,13,14]. In typical motile CFs, the main outer doublets complexes are dynein motors (outer and inner dynein arms, ODA and IDA), nexin–dynein regulatory complexes (N-DRC), and radial spokes (RSs) (of note, RSs are missing in mammalian nodal cilia) (for review see [14]). Additionally, typical motile CFs also contain so-called central apparatus (CA), composed of a pair of single microtubules connected by a bridge-like structure and anchoring numerous multiprotein complexes called projections [15,16] (Figure 1).

It is postulated that signals initiated at the CA are transmitted through mechano-chemical interactions between CA projections and RS heads to the RSs, and next to N-DRC and dyneins [17]. In consequence, the activity of the dynein motors causes sliding of the neighboring doublets at the specific sites within the axoneme, resulting in characteristic CF bending [17,18,19]. Thus, CA can play the role of a “kickstarter”, initiating the movement of the ciliary “nanomachine”. The site-restricted activation/inactivation of the dynein arms requires strict cooperation between different motile cilia structures to ensure coordination of intraciliary signals both along the cilium length and at cilium circumference. As a result, a complex, two-phases CFs beating pattern composed of power and recovery strokes is formed, ensuring efficient propelling of the fluid surrounding a ciliated cell.

Dysfunction of motile CFs, resulting in altered beating pattern and beating frequency, causes rare human genetic disorders, including primary ciliary dyskinesia (PCD), normal pressure hydrocephalus (NPH), and different forms of male infertility (asthenozoospermia, teratozoospermia, MMAF) [5,20,21].

Although numerous data suggest that CA is essential for the generation and regulation of motile CF beating, our knowledge of its composition and functioning is fragmentary. Here, we present a systematic review on CA, including the newest data concerning CA proteomics, analysis of its functioning, and human disorders caused by the mutations in genes encoding CA proteins.

## 2. Ultrastructure and Biogenesis of Central Apparatus

In typical motile cilia, the central apparatus consists of two thirteen-protofilament microtubules, called C1 and C2, which are docking sites for numerous protein complexes called projections (Figure 1) (of note, in non-typical motile nodal cilia, variation of the number of CA microtubules exist, and can vary from 0 to 4 [22]). The CA microtubules range from the transition zone (MTs minus-end) to the cilium tip (MTs plus end). At the distal cilium end (tip), the CA microtubules are longer than outer doublet MTs and are connected with the distal structure. Its organization may differ in various organisms (recently extensively reviewed in [23]).

**Figure 2:**
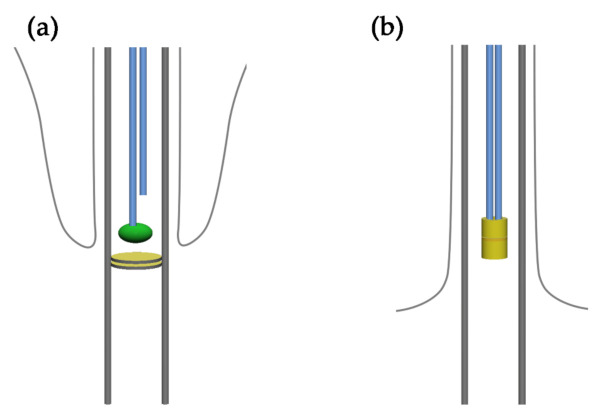
Docking of the CA microtubule minus-end: (**a**,**b**) a schematic representation of the basal body and proximal part of the cilium in a ciliate and green alga (**a**) in ciliates only one of CA microtubules is attached to the axosome (green oval), while the other starts more distally, yellow discs represents plates of the transition zone [46]; (**b**) both central microtubules are attached in the tube/central cylinder (yellow tube) in *Chlamydomonas* [24].

In contrast to outer doublets, CA microtubules do not originate from the basal body, but above it, in the distal region of the transition zone. Most frequently, the minus-end of one of two CA microtubules is capped [24] and embedded within a structure called basal plate in animals [25,26] and some protozoan parasites [24,27] or axosome in ciliates [28,29,30]. The other CA microtubule starts more distally and is uncapped (Figure 2A). In contrast, in *Chlamydomonas*, both CA microtubules are capped and attached to the central tube (also called central cylinder) [24,31] (Figure 2B). It was suggested that CA microtubule minus-ends capping and embedding is an organism-specific process [24]. Little is known about posttranslational modifications of tubulin in CA microtubules and the effect of these modifications on CA microtubule assembly, stabilization and functions. Recent studies suggest that similar to A-tubules of peripheral doublets, CA tubulin undergoes the majority of typical axonemal modification, including glycylation, and α-tubulin-specific modifications: acetylation, detyrosination, and Δ2, while glutamylation is less abundant [32,33].

The process of CA assembly is poorly characterized. Up to now, it was shown that lack of katanin, the heterodimeric microtubule severing complex, composed of the catalytic p60 and regulatory p80 subunits, is indispensable for the formation of the central apparatus in *Chlamydomonas* (*pf15*, p80 [34], *pf19*, p60 mutants [35]) and in *Tetrahymena* [36]. Lack of katanin subunits results in the formation of CA-less paralyzed CFs. The role of katanin in CA formation in mammalian CFs was not yet determined. Mutations or knockdown of Katnal1, a mammalian ortholog of p60, alters beating pattern and reduces the beat frequency of ependymal cilia and sperm cell flagella, causing symptoms typical for ciliopathies, including left-right asymmetry defects and hydrocephalus [37,38]. However, whether a reduction of Katnal1 affects CA was not determined. The entire CA is also frequently lost in *Chlamydomonas pf20* mutant (see below) [39,40] and in mammals with mutated Fu/STK36 kinase or Spef1 protein [26,41,42,43] (see below). Additionally, in a ciliate *Tetrahymena* [44] and *Drosophila* sperm cells [45], CA was not assembled in certain β-tubulin mutants.

In growing CFs, the CA assembly is initiated at the transition zone region and progresses toward the CF distal part, as was shown in *Chlamydomonas* [47,48], suggesting that structures located in the proximal part of the cilium (as basal plate, axosome or central tube) could be involved in CA microtubule nucleation. Accordingly, γ-tubulin was detected within the *Chlamydomonas* central tube [49], while in *Trypanosoma,* the RNAi-dependent depletion of γ-tubulin or γ-tubulin-small-complex proteins, GCP2 or GCP3, resulted in the formation of CA-less flagellum [50,51]. Moreover, basalin, a *Trypanosoma*-specific component of the basal plate, was also shown to be necessary for the formation of this structure and CA [52], further supporting the role of the CF proximal structures in CA assembly.

Interestingly, CA formation was initiated in the full-length flagella of katanin CA-less mutant once they were mated with wild-type cells. In fused cells (dikaryons) and in the presence of katanin (from wild-type partner), the nucleation of the CA structure is initiated in sub-distal parts of the CFs [48], but the mechanism of this process needs further clarifications.

**Figure 3:**
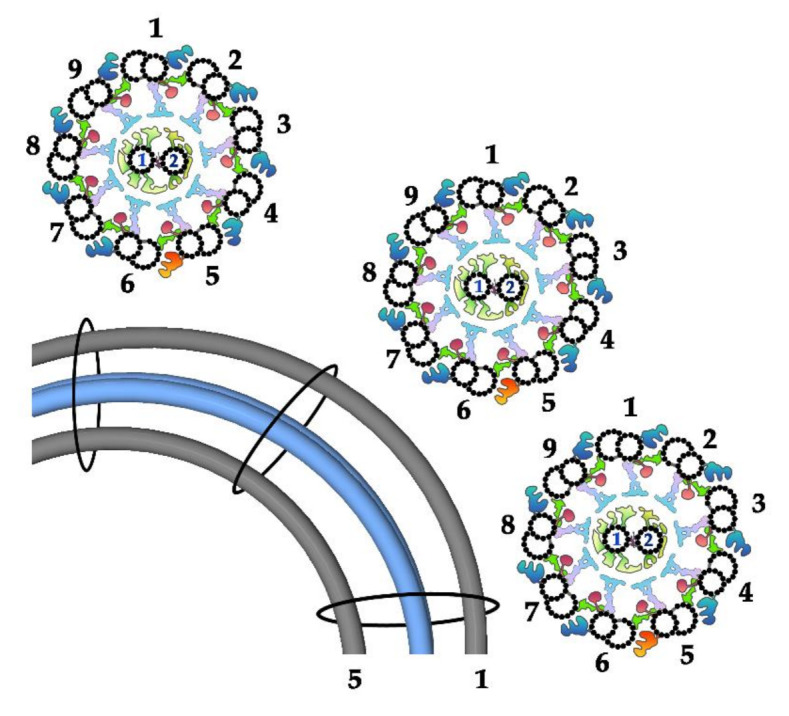
Fixed position of CA microtubules in *Strongylocentrotus* CF: scheme showing bent CF in longitudinal view with outer doublets number 1 and 5 (gray) and CA microtubules (blue) and in three cross-sections: before, at and after the bend (as shown on the longitudinal view of the cilium). Note that CA microtubules run parallel to each other along the entire cilium length and that their position is constant in relation to the outer doublets (each projection interacts with RS of the same outer doublet at the whole CF length). The position of the outer doublets and CA with respect to the bend, according to [60].

**Figure 4:**
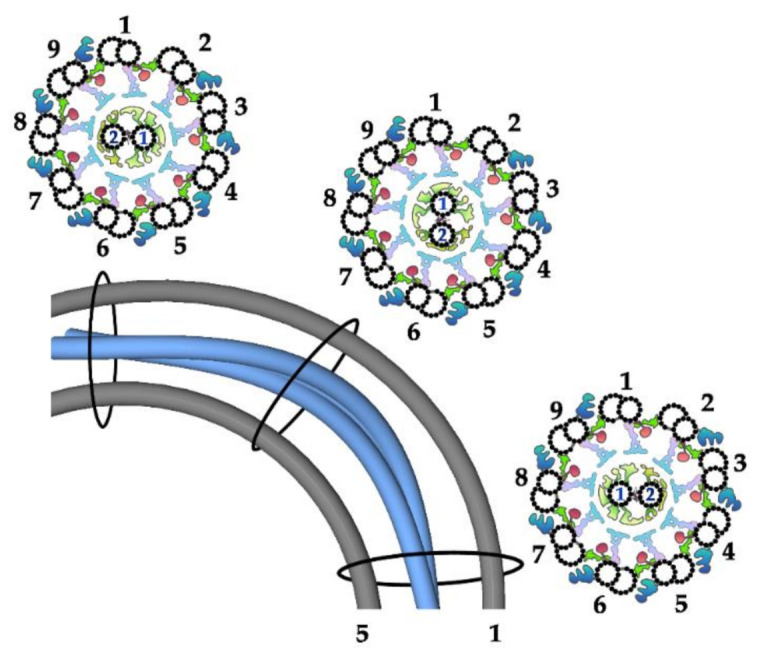
Twisted position of CA microtubules in *Chlamydomonas* CF: scheme showing bent CF in longitudinal view with outer doublets number 1 and 5 (gray) and CA microtubules (blue) and in three cross-sections: before, at and after the bend (as shown on the longitudinal view of the CF). Note that position of the CA in relation to the outer doublets changes (each projection interacts with RS of different outer doublets, depending on the position in relation to the bend). The position of the outer doublets and CA with respect to the bend, according to [60].

In animals and parasitic protozoa, CA microtubules were shown to have a fixed position with respect to the outer doublets and, in consequence, to the plane of the bend, with both CA microtubules facing the bend [53,54,55,56] (Figure 3). As a consequence, each C1 and C2 projection always interacts with RSs of the same outer doublet. In other protists studied, including *Paramecium* [57] and *Chlamydomonas* [58], it was shown that CA microtubules are twisted in a left-handed helix and the twist position changes following the shift of the CF bend. Accordingly, one of the CA microtubules (C1 in *Chlamydomonas* [58]) faces a convex (outside) and another (C2 in *Chlamydomonas*) a convey (inside) side of the bend [12,58] (Figure 4). This is probably true in the case of *Tetrahymena*, based on the analysis of cilia cross-sections of neighboring oral ciliary rows, which are in different phases of metachronal wave [59]. A shift of the CA twist position to accommodate it to the bend position requires rotation of the CA. Therefore, in this case, rather than interacting with RS of the specific outer doublet, each projection can interact with numerous outer doublets, depending on the position with respect to the bend (Figure 4).

The mechanism of CA rotation remains nearly unknown. It was shown that in *Chlamydomonas*, the twist of the CA microtubule is its intrinsic character. Moreover, the shift of the twist position within the CF is a passive process, driven by bend propagation, being independent of interactions with RSs [61]. In contrast, in *Paramecium,* twisting of CA microtubules seems to be an active process since, in paralyzed cilia, it is not observed [57]. Importantly, this diversity in CA position with respect to the outer doublets (fixed vs. rotating) could reflect differences in regulatory pathways governing the formation and propagation of CF bending [58].

## 3. Composition of Central Apparatus

Efforts done during the last 40 years revealed the identity of 22 *Chlamydomonas* CA proteins distributed within different projections [40,62,63,64,65,66,67,68,69], the majority of which appeared to be evolutionarily conserved. In this context, not only the structure of CA seems to be similar in evolutionarily distant organisms, but also its composition.

**Figure 5:**
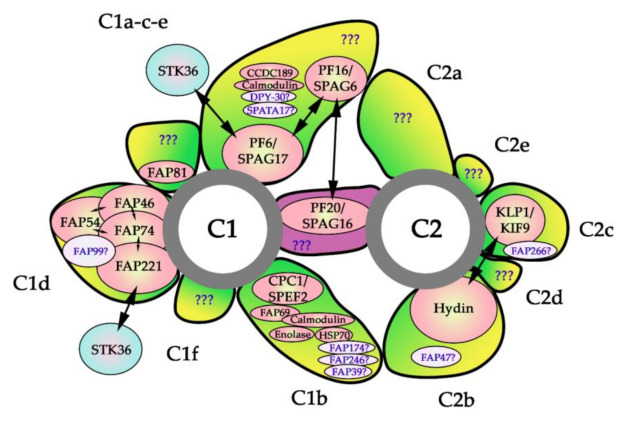
Predicted localization of the evolutionarily conserved subunits of the CA. C1 and C2 —central microtubules 1 and 2, respectively. The experimentally confirmed CA components are represented by peach ovals, putative subunits are shown as light pink ovals. Blue ovals represent protein transiently or weakly associated with CA; two-way arrows mark experimentally confirmed interactions between CA subunits. The question marks in the bridge and projections indicate putative unidentified subunits. See the text and Table 1 for details.

However, two recent proteomic studies of *Chlamydomonas* flagella led to the identification of approximately 40 novel candidate CA proteins [70,71], of which only 15 have human ortholog: FAP81/DLEC1, DPY30, FAP76/CCDC180, FAP305/SPATA17 (all four assigned to C1a-c-e projection), FAP246/LRGUK, phosphoglycerate mutase (both assigned to C1b), FAP99/CFAP99 (assigned to C1d projection), FAP47/CFAP47 (assigned to C2b), FAP266/RSPH10B (assigned to C2c-d), FAP65/CCDC108, FAP70/CFAP70, FAP147/AMAP-1 (all three assigned to C2, but not to a specific projection), FAP194/SPAG6, DIP13/SSNA1 and EF-3/ABCF3 (all three not assigned to specific CA microtubule) (Table 1, Figure 5) The remaining newly identified proteins appear to be green algae-specific. Therefore, there is still a large gap in our knowledge of potential components of CA outside this systematic group. Further studies are necessary to reveal which proteins substitute *Chlamydomonas*-specific CA subunits in CFs of other organisms.

Significantly, the components of C1e, C2a, and C2e are still unknown, whereas proteins forming other projections are much better characterized.

### 3.1. Bridge

The protein composition of the so-called bridge-like structure extending between C1 and C2 microtubules remains largely unknown. Up to now, PF20/SPAG16 is the only protein shown to localize between two central microtubules in *Chlamydomonas* flagella and, thus, likely being a component of the connecting bridge [40]. PF20 is an evolutionarily conserved, approximately 66 kDa protein, with several WD-repeats within its C-terminal part [40]. *Chlamydomonas pf20* mutant cells have paralyzed CFs, and upon isolation, they lack either both (73%) or one (27%) central pair microtubules (of note, in the same study, CA defects, although less frequently (~25%), have also been observed in isolated wild-type CFs) [39,40]. Thus, most likely, the bridge stabilizes CA. The phenotype of *Trypanosoma* mutants with a reduced level of PF20 (RNAi) seems to support this supposition. Flagella of *PF20* mutant cells are nearly paralyzed, but surprisingly, 80% of them have both CA microtubules (but frequently misoriented), and the remaining 20% miss only one CA microtubule [91]. The stability of the CA in this mutant could be explained either by the presence of residual PF20 protein, presence of the paraflagellar rod, a *Trypanosoma*-specific flagellar structure contributing to flagella stability, or fixation method used (entire cells were fixed for TEM analysis).

In mammals, *PF20/Spag16* gene encodes two proteins, a 71 kDa Spag16L (large) and a 35 kDa Spag16S (small). The latter corresponds to the C-terminal part of Spag16L, containing seven WD-repeats. The WD-repeat containing fragment can bind to another CA protein, PF16/Spag6 [92], indicating that both Spag16L and Spag16S could interact with this protein.

Spag16L is expressed in motile CF-forming organs, including testis, oviduct, and brain [93,94]. However, the lack of Spag16L seems to significantly affect only sperm cells, which in knockout males frequently have paralyzed CFs (without apparent changes in their ultrastructure), while the epithelial motile CFs seem unaltered [73]. Indeed, the beating frequency of the respiratory cilia in Spag16L mutant mice is only slightly reduced [77].

Spag16S is expressed exclusively in testes [93], where it localizes within the cytoplasm and nucleus of male germ cells before their maturation (of note, Spag16S is present in CF-forming elongating spermatids [73], but its precise subcellular localization was not determined). Interestingly, Spag16S enhances the expression of Spag16L [93].

The targeted disruption of exon 11, and thus elimination of both Spag16 variants, in embryonic cells subsequently injected into blastocysts, yield infertile chimeric male mice [72]. The mature sperm cells in these chimeras were much less numerous and showed many abnormalities, including bend tail with abnormal motion, lack of the CA (~30% of the cross-sections) and disorganization of outer flagellar doublets and outer dense fibers [72]. In mammalian sperm cell flagella, outer dense fibers enhance the stability of the entire axoneme [95,96]. Thus, their disorganization may indirectly contribute to the axoneme destabilization, including CA.

These results suggest that Spag16 plays an important role during germ cell development and maturation. No abnormalities were observed in chimera bronchial cilia, indicating that CF-related Spag16 functions are most likely restricted to sperm cells. Importantly, the presence of Spag16S in the nucleus and its interactions with a nuclear protein, MEIG1, led to the hypothesis that Spag16 proteins play the additional role(s) in the process of spermatogenesis besides its structural function in the axoneme [72,93].

The experiments conducted in mice revealed surprising differences in the phenotypic and structural alterations in respiratory cilia and sperm cells flagella as well as between mammals and *Chlamydomonas* CFs caused by PF20/SPAG16 deletion. Because *Chlamydomonas* flagella lack additional supporting structures, such as paraflagellar rod in *Trypanosoma*, one could speculate that the lack of CA in *Chlamydomonas pf20* mutants could be a secondary effect caused by forces generated during flagellum bending. It was also proposed that the differences in ciliary waveform (classical effective and recovery strokes in *Chlamydomonas* and a ”snake-like” movement of mammalian flagella) could explain higher resistance of CA in sperm cells flagella assembled by *spag16* mutant [97]. On the other hand, it cannot be excluded that CFs in mammals contain proteins redundant with Spag16. The presence of such protein(s) would explain why the ultrastructure and beating of epithelial CFs are not affected in Spag16 mutant mice.

In humans, the mutation in a single *SPAG16* allele did not cause PCD symptoms or infertility [98], while individuals carrying mutations in both alleles were not reported.

### 3.2. C1a-c-e Projections

C1a projection of the estimated size of 1.1–1.7 MDa, C1c (1.9–2 MDa), and C1e projection (0.2–0.5 MDa) [99], previously described as a sheath material [100] form a supercomplex, assembly, of which depends upon the presence of PF16/Spag6 protein (see below) [78].

In *Chlamydomonas* PF6, together with evolutionarily conserved proteins, calmodulin, two orthologous FAP114/CCDC189 and FAP119/CCDC189, and two nonconserved proteins, were reported to form a C1a projection [62] (Table 1). Recently, also PF16/Spag6 and other three conserved subunits, FAP81/DLEC1, MOT17/SPATA17 and DPY30 (Table 1), as well as additional algae-specific proteins, were shown to build C1a-c-e supercomplex [70,71,78]. The functions of the majority of the components of this supercomplex in CF motility are poorly understood. The increasing number of data suggests that besides ciliary roles, C1a-c-e components are also involved in spermatogenesis (see below), but it is not known if they function as complex or have independent functions in this process.

PF6/Spag17 and PF16/Spag6 are the most extensively studied proteins of the C1a-c-e supercomplex. The *Chlamydomonas* PF6 and mammalian SPAG17 are large, ~250 kDa, proteins sharing similarity mainly within the C-terminal part. PF6/Spag17 is essential for the formation of C1a projection and CF motility in both *Chlamydomonas* [101] and mammals [74]. Flagella of the *Chlamydomonas pf6* mutant show limited jiggling motion, enabling only occasional forward movement of the cell [101,102]. Interestingly, expression of the PF6 truncations, encompassing sub-C-terminal part restores assembly of at least a part of C1a projection and rescues *pf6* mutant motility. The rescue efficiency depends upon the size of the truncated protein. In contrast, *pf6* truncations devoid of the sub-C-terminal part are unable to restore flagella beating [103].

Spag17, an ortholog of *Chlamydomonas* PF6, localizes within CA, as was shown in mouse sperm cells [104] and plays a role in the formation of CA projection [74]. Mice with disruption of *Spag17* gene die within few hours after birth because of respiratory distress and hydrocephalus. Respiratory motile CFs are formed at the normal density but are immotile or occasionally move in an uncoordinated fashion. TEM analysis showed that most frequently, CFs lack the C1a projection and that CA microtubules are more distant from each other than in wild-type cilia. In some cilia (~23%), one or even both central pair microtubules are missing [74].

Deletion of Spag17 in mice also affected the formation of sperm cells. Surprisingly, the majority of studied axonemes showed normal ultrastructure. Instead, affected males produced a low number of sperm cells, and mature sperm cells had abnormal head shape and reduced tail length, indicating that Spag17 is involved in spermatogenesis [105]. It was also reported that mutation resulting in the formation of shorter, C-terminus-deprived protein caused PCD and infertility symptoms in mutant mice. The respiratory CFs beat with altered waveform and sometimes frequency and showed some ultrastructural defects. Moreover, in mutant testes, the mature sperm cells were not formed, suggesting that the truncated Spag17 was not sufficient for spermatogenesis [106]. In contrast, sub-C-terminally located point mutation of SPAG17 in a human patient caused asthenozoospermia -related infertility, but not PCD [76].

Intriguingly, Spag17 also regulates the formation of the primary cilia, at least in a subset of cell types. Osteoblasts and chondrocytes isolated from Spag17-deficient mice form shorter primary cilia and with lower frequency. This phenotype could explain defects in bone formation and mineralization in *Spag17*-null mutant [75].

PF16/SPAG6, an approximately 60 kDa armadillo repeats-containing protein [66], plays a crucial role in the formation and/or stability of the C1a-c-e supercomplex [78]. Flagella assembled by *Chlamydomonas pf16* mutant lack the entire C1a-c-e supercomplex [78] and are nearly completely paralyzed with only ciliary tips twitching and occasional formation of ciliary waveform [101].

In mice, knockout of *Spag6* causes symptoms typical for PCD, but ciliary defects are less severe than those observed in *Chlamydomonas*. Respiratory CFs in mutant mice seem to have normal ultrastructure and only beat with reduced frequency [77]. However, the number of CF is lowered, and the orientation of the basal feet is affected in respiratory epithelium and ependyma [77], suggesting a role of Spag6 in cilia biogenesis. On the other hand, *Xenopus* embryos with morpholino-induced knockdown of Spag6 assemble a normal number of CF. Yet, similar to mice, they show some ultrastructural defects and their motility and orientation are affected [107].

Spag6-deficiency also causes male infertility. Mostly, the sperm cells produced by Spag6 knockout males are hardly motile, and nearly half of them have structural abnormalities (headless, short tail, lack of CA, disorganization of the outer doublets and outer dense fibers) [108].

When overexpressed, Spag6 was shown to colocalize with cytoplasmic microtubules in non-ciliated and primary cilia-forming cells [94,104], suggesting the affinity of Spag6 to microtubules. This result, together with data suggesting the involvement of Spag6 in the regulation of cell morphology, proliferation, and migration (including neurons), as well as the formation of microtubule-based specialized structures, such as primary cilia, neurites, and immunological synapse [109,110,111,112] hint that Spag6 may play broad microtubule-related functions.

Biochemical studies suggest that PF16/Spag6, PF6/Spag17, and the bridge protein, PF20/Spag16, interact and likely localize interdependently. PF16/Spag6 interacts with PF6/Spag17 via the C-terminal part of PF6/Spag17 [104] and with WD-repeats of PF20/Spag16 [92]. Moreover, when co-expressed with PF16/Spag6, PF6/Spag17 re-locates from the cytoplasm to cytoplasmic microtubules [104]. These data are in agreement with the analysis showing that in *Chlamydomonas pf16* flagella and mouse *PF16/Spag6* knockout epididymal sperm cells, the level of PF6/Spag17 is reduced (one specific isoform containing C-terminal part of the protein, in the case of mice) [78,104]. However, in contrast to *Chlamydomonas*, in mice also the level of PF20/Spag16 is lowered in PF16/Spag6-null sperm cells [104]. On the other hand, in testes of PF20/Spag16 chimeras, the level of PF16/Spag6 remains unaltered [72], suggesting that Spag6 targets Spag16 to the CFs, but not vice versa.

The knowledge regarding functions of the remaining components of the C1a-c-e supercomplex is sparse. Bulls with a homozygous null-mutation in an ~30–40 kDa, coiled-coil domain-containing protein, CCDC189, produce sperm cells with flagella of normal ultrastructure but nearly immotile [79]. Furthermore, knockout of *FAP81*/*Dlec1* in mouse causes male infertility, but due to aberrant spermatogenesis [80] manifested by the production of a low number of mature sperm cells with abnormal morphology, including head shape malformation and short flagellum [80]. In contrast, CFs of *Chlamydomonas fap81* mutant lack C1c and e projections and beat with reduced beating frequency, altered waveform and asynchrony [78]. Interestingly, FAP81/DLEC1 protein could also play non-ciliary functions, as it was identified as a tumor suppressor [113].

Finally, some data suggest that MOT17/SPATA17, a small ~30 kDa protein, can also be involved in the process of spermatogenesis, as overexpression of human SPATA17 in mouse testes increased germ cell apoptosis [114]. SPATA17 contains IQ motifs; thus, it can be involved in calcium signaling.

### 3.3. C1b and C1f Projections

C1b (estimated MW 1.3–1.9 MDa) is located at the opposite side of the C1a and near C1f (1.1–1.6 MDa) [99], earlier described as a sheath extending between C1b and C1d [100]. Flagella of *Chlamydomonas* C1b-less mutant (*cpc1*, see below) lack both C1b and C1f projections [100]. Thus, C1b and C1f likely form another C1 supercomplex. Early studies of *Chlamydomonas* flagella showed that C1b/C1f is likely composed of several evolutionarily conserved proteins, CPC1/SPEF2, FAP69/CFAP69, enolase (ENO), and HSP70 [63,69]. Additionally, comparative proteomic analysis indicates that, besides several algae-specific subunits (Table 1), conserved FAP246/LRGUK, phosphoglycerate mutase (PGM), FAP39/ATP2B3, and FAP174/MYCBP could be components of C1b/C1f [70,71]. Of note, ENO, HSP70, and FAP174 were suggested to associate also with other ciliary structures [69,71].

CPC1 and its mammalian ortholog SPEF2 are large, ~200–250 kDa proteins, containing CH (calponin homology) domain, adenylate kinase domain, and EF-hand motif [63]. Flagella assembled by the *Chlamydomonas cpc1* mutant exhibit normal waveform, but their beating frequency is only half of that observed in wild-type cells [100]. Ultrastructural analysis showed that mutant flagella lack C1b/f and frequently also C2b projections, suggesting that C1b can interact with and stabilize C2b [100].

In mice [81,82] and humans [82,115], mutation of *Spef2* causes PCD symptoms and male infertility. The ciliary defects in murine Spef2 mutants are similar but not identical to those observed in *Chlamydomonas*. Although the beating frequency of the respiratory cilia is slightly (~20%) reduced, the cilia ultrastructure was described as normal [81,82]. However, it cannot be excluded that some minor ultrastructural defects remained undetected using classical TEM. In contrast, the morphology, ultrastructure, and motility of sperm cells are severely affected in *Spef2* mouse mutants. A lack of CA was observed at the early-stage of spermatogenesis, while in maturing sperm cells, all flagellar structures were highly disorganized [81,82]. Similar sperm cell defects were observed in pigs [116] and humans [117,118,119] carrying *SPEF2* mutation.

In patients with PCD caused by *SPEF2* mutation, the respiratory cilia did not show apparent ultrastructural abnormalities (although it seems that there is a gap in CA structure corresponding to the C1b position, Figure 4C in [82]).

The potential partners of Spef2 and thus, the potential subunits of C1b/C1f supercomplex were identified in *Chlamydomonas* as components of the 16S complex. These are algae-specific FAP42 and three already mentioned evolutionarily conserved proteins, FAP69, enolase, and HSP70 [69]. Levels of all these proteins in flagella were significantly reduced in *Chlamydomonas cpc1* mutants [69]. Accordingly, CFAP69 was detected in sperm cell tails of healthy donors but not in sperm tails of SPEF2-deficient individuals [117].

In mammals, Spef2 can also interact with IFT20, an anterograde intraflagellar complex protein [120]. Likely, Spef2 regulates IFT20-related intraflagellar transport, since in Spef2 knockout mice this process is delayed [121]. Moreover, Spef2 was also shown to have a primary cilia-related function(s) during osteoblast differentiation, but its role in this process is poorly understood [122].

Enolases and HSP70 play numerous functions in a variety of cell organelles, and their role in motile CF is not clear. In mouse deletion of testis-specific enolase, Eno4 leads to male infertility related to sperm cell abnormalities. Sperm cell morphology in Eno4 knockout resembles that of Spef2 knockout; however, the ultrastructural defects of sperm tail were rather related to sperm accessory structures (fibrous sheath) than to axoneme itself [83]. During sperm flagella lysis, the majority of Eno4 remains within the insoluble fraction. Moreover, Eno4 interacts with AKAP4, a component of the fibrous sheath. Thus, it is highly probable that Eno4 is located within these accessory structures rather than within the CA. However, most likely, Eno4 is not the only enolase within sperm cells [83] and therefore, it is tempting to speculate that another enolase (most probably Eno1 [83]) participates in local ATP level regulation within CA of the sperm cells. Hence, so far, the role of Eno1 in motile CF has not been addressed.

Although the role of HSP70 was broadly studied, little is known about its function in motile CFs. In *Chlamydomonas,* it was shown that HSP70 participates in the regulation of flagellar microtubule assembly, probably playing a role in protein folding, transport, and repair as in other systems [123,124,125].

FAP69/CFAP69 is an ~100–120 kDa armadillo repeats-containing protein [126]. The knowledge about FAP69/CFAP69 functions is limited, but recent studies show that it is important in sperm cells and olfactory neurons that form modified cilia as sensory organelles [84,126].

CFAP69 was shown to be located exclusively in the proximal part of sperm flagella (midpiece) and to be required for flagellar localization of Spef2 [84,127]. Knockout of Cfap69 in mice and mutations of CFAP69 in humans cause the formation of abnormal sperm cells that morphologically resemble that of Spef2 knockout. Interestingly, while in mouse mutants, all flagellar structures are severely disorganized, in human sperm cells, normal outer doublets are formed, but the whole central apparatus is missing [127]. However, as in the case of Spef2, Cfap69 has multiple alternative splicing isoforms, which could be differentially expressed in various CF-forming tissues. Therefore, mutations that affect sperm cell functions may not be deleterious for motile epithelial cilia. Until now, the defects in functions of epithelial CFs were not reported in individuals carrying mutations in *CFAP69* or in Cfap69 mutant mice [84,127].

In *Chlamydomonas*, lack of the ~120 kDa FAP246 slightly reduces cells swimming rate [70]. The mammalian ortholog, LRGUK, shares similarity with FAP246 only within N-terminal, LRR-containing fragment, but not within the C-terminal fragment containing guanylate kinase domains. During spermatogenesis, LRGUK plays a role in the formation of acrosome and elongation of CF [85,128]. A significant decrease of one (of three) Lrguk isoforms in mice caused improper attachment of the flagellar basal body to the plasma membrane and subsequently lack of the axoneme elongation [85]. Accordingly, Lrguk was shown to bind proteins that are known to be involved in the process of spermatogenesis: HOOK1, HOOK2, HOOK3, RIMBP3, and KLC [128,129].

### 3.4. C1d Projection

C1d is the largest CA projection (estimated MW of 2.4–2.6 MDa), although it does not extend as close to the radial spokes as C1a and C1b [99]. The studies in *Chlamydomonas* indicate that evolutionarily conserved proteins, FAP46, FAP54, FAP74, FAP221, FAP297/WDR93, calmodulin, and likely FAP99 [64,65,71] build the C1d projection.

Similar to C1a projection, mutations in mammalian C1d subunits are associated with male infertility caused by sperm cell malformations. However, mammalian C1d-mutants also develop PCD, indicating that aberration in this projection strongly affects the functioning of motile CFs assembled by different cell types.

FAP221 and its mammalian ortholog, CFAP221/Pcdp1 (primary ciliary dyskinesia protein 1), are ~100–110 kDa proteins containing a calmodulin-binding domain. Thus, FAP221 orthologs could be involved in calcium-dependent regulation of ciliary functions. In mice, lack of Cfap221/Pcdp1 causes PCD and male infertility without any obvious ultrastructural abnormalities in the respiratory cilia [87]. A PCD-causing mutation in *CFAP221* was also identified in humans; in the affected individual, the respiratory cilia beat with normal frequency but abnormal waveform, forming rotatory-like rather than planar movement [88].

FAP54/CFAP54, an ~300 kDa protein, can bind calmodulin [65]. In mice with Cfap54 depletion, the respiratory cilia lack C1d projection and beat with reduced rate causing the development of respiratory symptoms (PCD) [86]. Additionally, Cfap54-depleted males are infertile due to defects in spermatogenesis and the formation of short-tail sperm cells.

FAP46, an ~300 kDa protein, and FAP74, an approximately 200 kDa protein, were analyzed only in *Chlamydomonas*. Flagella of *fap46* mutant lack C1d projection, beat asynchronously, with reduced frequency and changed waveform [65].

Flagella of FAP74 knockdown are depleted of both C1d and f projections. In consequence, cells with FAP74 knockdown swim with flagella beating patterns similar to *fap46* mutant but with even lower frequency [65]. Thus, a decrease of FAP74 causes a more severe phenotype than lack of FAP46. Recently, point mutations in CFAP74 were identified in two patients as a cause of PCD and infertility related to short-tail and axoneme disorganization phenotype [130].

### 3.5. C2b Projection

The C2b projection is facing the C1b projection but is much smaller (approximately 1–1.1 MDa [99]). Based on the ultrastructural analyses of *Chlamydomonas* and mouse mutants, it appears that approximately 540 kDa protein, hydin (hydrocephalus associated protein), is the main subunit of C2b. Recent proteomic studies suggest that another ciliary protein, FAP47/CFAP47, could also form a part of C2b projection [71].

In *Chlamydomonas*, knockdown of HYD results in lack (about 30–40% of cells) or assembly of shorter (40% of the wild-type length) CFs, with residual hydin present most frequently in the proximal part of only one of them. Mutant CFs lack C2b and part of C2c projection, suggesting that besides C2b, hydin either builds a part of C2c or is required for its stabilization. Less frequently, also other abnormalities, such as a loss of one or both CA microtubules or outer doublet abnormalities, were observed [68]. These ultrastructural aberrations correlate with CFs paralyzed in an unusual CFs configuration. While in other *Chlamydomonas* CA mutants (*pf6*, *pf16*), CFs point away from the cell body (so-called “hands-up position”), in approximately 80% of *Chlamydomonas hyd* cells, one or less frequently, both CFs are positioned alongside the cell body (so-called “hands-down position”), indicating probable defects with the initiation of the recovery stroke [68]. In some *Chlamydomonas hyd* mutants, flagella could twitch (mostly the ones in “hands-up position”), with occasional very quick power and recovery strokes [68], likely due to the presence of the residual Hydin.

In *Trypanosoma*, similar to *Chlamydomonas*, depletion of hydin causes cell immotility, with some flagella lacking one or both CA microtubules. Regretfully, it was not described if, in CA-containing CFs, C2b and C2c projections are normally formed. Interestingly, in *Trypanosoma* hydin knockdown, the CA was frequently misoriented [131].

In mammals, mutations in hydin cause PCD-related phenotype. Hydin-deficient mice develop hydrocephalus [33,132,133], while in humans, respiratory distress was the main symptom [89]. In the respiratory CFs of both mice and humans, hydin deficiency resulted in a loss of C2b and absent or incomplete C2c projection [33,89]. Moreover, in mouse, C1b projection was frequently displaced or altered in shape [33] while in humans, C1b projection protein, Spef2, was reduced [115], suggesting that C1b and C2b projections are connected and/or that their stability is interdependent (which is also suggested by the reduction of C2b projection in C1b-less mutants, see above).

In contrast to *Chlamydomonas*, murine and human hydin-depleted epithelial CFs were able to beat, although with lowered amplitude (and frequency in mouse) [33,89]. On the other hand, in humans, approximately 90% of the hydin-deficient sperm cells had nearly or completely immotile flagella [89]. Such analysis was not possible for mouse sperm, as hydin-deficient animals died before reaching reproductive age [33].

In *hydin* mutant mice, similar as in PF16/Spag6 mutant, the basal bodies in ependymal ciliated cells are frequently misaligned [33], and individual cilia beat asynchronously. Lack of CFs synchrony was also observed in patient respiratory cilia suggesting that the presence of hydin is required for spatial cilia orientation [33,89].

In silico study suggests that hydin has four ASH domains. The biological significance of ASH is unknown, but it was hypothesized that the ASH domain mediates the targeting of CF proteins to the CA [68,134]. Accordingly, it was shown that the ASH domain of murine hydin could interact with Kif9 kinesin, an ortholog of C2c-associated KLP1 (see below), and that hydin is essential for the flagellar localization of Kif9 in mouse sperm [90].

### 3.6. C2c Projection

Identified in *Chlamydomonas* Klp1/Kif9 [67] is, so far, the only know component of the relatively small (0.4–0.5 MDa) C2c projection [99]. Klp1/Kif9 is a phosphoprotein of approximately 90 kDa belonging to the kinesin-9 family [67,135,136]. The biochemical analysis indicates that it can interact with microtubules in a kinase-like manner [67], but the role of KLP1/Kif9 within CFs is unknown.

The knockdown of KLP1 in *Chlamydomonas* causes a decrease of CF beating frequency related to the loss of a part of C2c and reduction or displacement of C2b projection. In contrast, in *Trypanosoma* knockdown of KIF9A, an ortholog of KLP1 reduced cell swimming rate, but the apparent ultrastructural changes in mutant flagella were not detected [137].

Until now, there is no evidence that mutations in Kif9 cause PCD in humans or mice. However, mouse males with Kif9 mutation have lower fertility, likely due to reduced sperm cell motility and abnormal flagellar waveform [90].

### 3.7. Mammalian Central Apparatus Proteins

#### 3.7.1. Stk36

Fu (Fused)/STK36 is an ~140 kDa serine-threonine kinase. In *Drosophila melanogaster* Fu is a part of the intracellular multiprotein hedgehog signaling complex (HSC) that mediates hedgehog (Hh) signaling (reviewed in [138]). In mammals, STK36 functions are not related to Hh signaling (which is mostly primary cilia-dependent). Rather, mammalian STK36 was shown to play a role in motile CF. In mouse oviduct and respiratory ciliated cells, Stk36-mCherry fusion protein localizes along the entire length of motile CFs, but not in immotile primary cilia of mouse embryonic fibroblasts [26]. Similar STK36 localization was observed in ciliated human respiratory cells [42]. Moreover, Stk36 interactions with PF20/Spag16 [26,41] and Cfap221/Pcdp1 [26] further support the hypothesis that Stk36 is a novel component of mammalian CA. On the other hand, STK36 was not detected in cilia of the respiratory cells obtained from patients with mutations in the radial spoke head proteins, RSPH1, RSPH4A, and RSPH9 [42]. Thus, STK36 could localize at the interface between radial spokes and CA projections [42] or its docking to CA could be stabilized by RSs.

In mammals, mutations of Fu/STK36 cause PCD symptoms [41,42]. In Stk36-knockout mouse, motile CFs beat with reduced frequency and amplitude [41], and up to 80% of the tracheal, ependymal, and oviduct cilia lacked the whole CA [26,41]. In humans, a mutation leading to premature stop codon positioned after the fragment encoding kinase domain also caused PCD, but a vast majority of the respiratory cilia of the affected individuals had normal ultrastructure [42]. It cannot be excluded that a truncated, kinase domain-containing STK36, with some activity, could be produced in this patient. It is tempting to speculate that Fu/STK36 kinase activity is sufficient for the formation or stability of the CA but insufficient for correct CF beating.

As in the case of depletion of other CA components, in both human patients and knockout mice, the basal bodies and CFs in respiratory epithelial cells are disoriented, and thus very likely, the ciliary synchronization is compromised [41,42].

Surprisingly, Fu/STK36 ortholog was not identified in the *Chlamydomonas* flagellar and CA proteomes [70,71,139], although such ortholog is encoded by the *Chlamydomonas* genome. However, it cannot be excluded that STK36 is present at a very low-level in *Chlamydomonas* CFs and therefore, it is difficult to detect.

#### 3.7.2. Spef1/CLAMP

Spef1 is an evolutionarily conserved protein with microtubule binding and bundling properties [140]. The vast majority of the murine ependymal cilia with Spef1 depletion (more than 80%) lacks the CA and exhibit a rotatory movement [43]. Murine Spef1, with a point mutation in the calponin homology domain disrupting its MT-binding properties, can be targeted to cilia but with lower efficiency than the wild-type protein. Moreover, the expression of this mutant form in Spef1-knockdown ependymal cells does not restore CA formation [43]. Thus, MT-binding and/or bundling activity seems essential for CA formation.

The immunofluorescence analysis showed that Spef1 likely localizes to the CA in ependymal cilia [43]. Surprisingly, although Spef1 was detected in the tails of murine sperm cells, it was not associated with the axoneme [141]. Moreover, in *Trypanosoma*, a Spef1 ortholog, TbSpef1, was shown to colocalize with a bundle of MTs supporting CFs (between the kinetoplast and flagellum attachment zone), but not within the CF itself [142]. Thus, it remains to be determined whether Spef1 is a component of the CA in other mammalian cell types and in other organisms and what is the exact role of this protein in the CA assembly and/or stability.

## 4. Central Apparatus Functions

The analysis of the CA-less mutants indicates that CA plays an important role in CF beating generation and regulation. Interestingly, the phenotypic outcome of the lack of CA can vary depending on the organism or even tissue type within the same organism. For example, CFs of CA-less *Chlamydomonas* mutants (*pf20*, *pf15*, and *pf19* mutants) are paralyzed [143] while murine CA-less cilia (STk36 knockout, Spef1 knockout) are motile, but move with reduced frequency, altered amplitude and waveform, and with a beating pattern frequently changed to rotatory-like. Moreover, comparison of CFs motility in different ciliated tissues of Fu/Stk36 mutant shows that tracheal CFs beat with ~35% of wild-type frequency and with very low amplitude, while oviduct CFs show ~50% of frequency decrease, but their amplitude is less affected than in tracheas [26,41].

The comparison of the phenotype of *Chlamydomonas* mutants lacking specific projection suggests that each of them could have its own specific, not interchangeable, functions in the regulation of CF beating. For example, lack of the whole C1a-c-e supercomplex (*pf16* mutant) results in nearly total CF paralysis (with two CFs stiffly extending from the cell body (so-called “hands up” position) [101] suggesting difficulties in the generation of the power stroke. Interestingly, loss of C2b (*hyd* mutant) also causes nearly total CF paralysis, but frequently one or two CFs extend along the cell surface (so-called “hands-down” position), suggesting problems in recovery stroke initiation and/or execution [68]. Thus, it is tempting to speculate that these two projections located on the opposite sides of CA could participate in the initiation or regulation of power and recovery strokes, respectively.

Loss of other projections is less severe. Lack of C1a (*pf6* mutant), the biggest part of the C1a-c-e supercomplex, results in “twitchy” CFs, moving with very low frequency and slightly abnormal waveform [101,102], while lack of C1c and e projections (*fap81* mutant) causes asynchronous beating, likely due to different frequencies and slightly modified waveform. CF with the elimination of C1e projection (*fap76* mutant) beat asynchronously, but with only slightly reduced frequency and normal waveform (see Video 3 in [78]). Loss of the whole C1b projection (accompanied by destabilization or lack of C1f and C2b, *cpc1* mutant) only decreases CF beating without affecting flagellar waveform [100], while CFs deprived of C1d move slower than wild-type and show abnormalities in waveform and flagella synchronization [64,65].

How CA regulates CF beating? The presence of proteins with functional domains and enzymes in different projections could suggest that particular projections are involved in specific signaling pathways. For example, C1a contains calmodulin, suggesting its involvement in calcium signaling [62]. Accordingly, analysis of CF isolated from CA-defective mutants showed that activation of dynein arms in the presence of high calcium concentration was affected in C1a-lacking flagella (*pf6* mutant), but not in C1b-lacking flagella (*cpc1* mutant) [144]. On the other hand, C1b was shown to contain enolase and possibly phosphoglycerate mutase [69,71], two enzymes involved in the glycolytic pathway of ATP production. Thus, C1b projection could play a role in local ATP level maintenance [63]. In fact, in permeabilized cell models, upon the increase of ATP concentration, the beating frequency of C1b-depleted flagella was restored to the wild-type level [63].

It is proposed that signals produced at the CA are transmitted to radial spokes by mechano-chemical pathways [17]. However, the nature of the interactions between RSs heads and CA projections and their influence on ciliary beating is still largely unknown. Oda and colleagues [19] showed that the immotility of *Chlamydomonas pf6* mutant lacking C1a projection could be partially suppressed by expression of RSP3, RSP4, or RSP6 with a tag attached to their C-terminal end. Such a tag is exposed toward the CA. The RSP fusion proteins were unable to rescue the slow-motility of *cpc1* mutant lacking C1b projection [19]. These results suggest that at least in the case of C1a projection, mechanical interactions with radial spoke head(s) would be sufficient to enable signaling between these two structures.

A recent study from the Vale group [145] suggests that the electrostatic forces could be involved in CA-RS crosstalk and regulation of cilia motility. The surface of the radial spoke head facing the CA is enriched in negatively charged amino acids. *Chlamydomonas* mutant cells with the negatively charged amino acids replaced by uncharged ones swam in spiral-like rather than straight paths, and CF beating frequency was reduced compared to wild-type.

## 5. Conclusions and Future Directions

Despite the recent significant progress, we are only at the beginning of the road to answer questions regarding structure, composition and role(s) played by the CA in the intricate process of cilia beating regulation.

Most information regarding the protein composition of the CA complex and its functions was obtained using *Chlamydomonas* as a model [70,71]. Surprisingly, a significant number of the identified proteins is algae-specific. Thus, it remains to be determined which proteins build the CA in other organisms, including humans. Further studies of the CA proteins, their functional domains, and posttranslational modifications would be an important step in the understanding of the processes taking place at CA.

The structural CA proteins can form a scaffold for enzymes that could be loosely or transiently associated with CA projections. Such enzymes could locally regulate the level of signaling molecules, energy sources (e.g., ATP) or modify CA subunits (e.g., phosphorylation/dephosphorylation). It was already shown that enolase [69], calmodulin-binding proteins [64,65,144], STK36 kinase [42], and phosphatase [71,146] are likely associated with CA

Although it is widely accepted that the signals regulating cilia beating originate at the CA and are transmitted via radial spokes to dynein arms, the nature of these signals is still unclear. Available data suggest that mechanical or mechanochemical signals can be transmitted by transient physical interactions between radial spokes and C1a projection [19]. This raises a series of questions. Do all of the CA projections participate in signal transduction? If yes, what is the nature of the transduced signals? Which of the components of particular projections interact with radial spoke proteins? Do these interactions involve second messengers?

With few exceptions (e.g., *Chlamydomonas*), each outer doublet axonemal unit contains three radial spokes that slightly differ in their architecture [147] and, thus, possibly, also protein composition. Likely, all radial spokes participate in such interactions, but are these random interactions or do particular spoke interact with certain CA projections?

Finally, the mechanism behind the CA assembly and the role of the IFT particles in this process are unclear. In addition, we can only speculate about the significance of the microtubule severing protein, katanin, STK36 kinase, SPEF1 or tubulin modification in this process.

Likely, further analysis and use of sophisticated experimental tools will help researchers to answer these and many other questions and dissect the molecular mechanisms behind this ciliary kickstarter activity.

## Figures and Tables

**Table 1:** Confirmed and putative CA proteins.

Projection	*Chlamydomonas*	Mammalian Homolog	Mass (kDa)	Domain Composition	Disorders		References
					Mammals	Human	
Bridge	PF20	SPAG16	67	WD domains	Infertility	-	[40,72,73]
C1a-c-e	PF6	SPAG17	250	Alanine–proline rich	PCD/hydrocephalus, male infertility, abnormal bone formation	Asthenozoospermia	[62,74,75,76]
	PF16	SPAG6	60	Armadillo repeats	PCD/hydrocephalus, male infertility,	-	[77,78]
	FAP114	CCDC189	32	Coil-coiled motifs	Asthenozoospermia	-	[62,79]
	FAP119		34				
	FAP81	DLEC1	172	ASH	Male infertility	-	[78,80]
	MOT17	SPATA17	28	IQ motif	-	-	[78]
	DPY-30	DPY-30	11	DPY-30	-	-	[78]
C1b-f	CPC1	SPEF2	265	Adenylate kinase domain, EF-hand motif	PCD/hydrocephalus, male infertility	PCD, male infertility	[69,81,82]
	Enolase	Enolase	56	-	Male infertility	-	[69,83]
	HSP70	HSP70	78	-	-	-	[69]
	FAP69	CFAP69	115	Armadillo Repeats	Male infertility	Male infertility	[69,84]
	FAP174	MYCBP	10		-	-	[70,71]
	FAP246	LRGUK	32	LRR, TGC, EF-hand motifs	Male infertility	-	[70,71,85]
	FAP39	ATP2B3/ ATP2B4	101	Calcium-transporting ATPase	-	-	[70]
C1d	FAP46	CFAP46	289	-	-	-	[64]
	FAP54	CFAP54	318	-	PCD/hydrocephalus, male infertility	-	[64,86]
	FAP74	CFAP74	204	ASH domain	-	-	[64]
	FAP221	CFAP221/PCDP1	100	Calmodulin-binding domain	PCD/hydrocephalus, male infertility	-	[64,87,88]
	FAP297	Weak homology to WDR93	87	WD domain, calmodulin-binding domain	-	-	[65]
	FAP99	CFAP99	90	Neuromodulin family	-	-	[70,71]
C2b	Hydin	Hydin	540	ASH domains	PCD/hydrocephalus	PCD, asthenozoospermia	[33,68,89]
	FAP47	CFAP47	310	ASH domain, CH domain	-	-	[70,71]
C2c/d	Klp1	KIF9	90	Kinesin motor domain	Subfertility	-	[67,90]
	FAP266	RSPH10B	22	MORN repeat	-	-	[70,71]
Mammals-specific	CHLRE_06g290900v5	SPEF1	23	Coil-coiled motifs, calponin homology	-	-	[43]
	CHLRE_09g412750v5	STK36	144	Serine-threonine kinase	PCD/hydrocephalus	PCD	[41,42]

## Data Availability

Not applicable.
